# Evaluating the direct effects of childhood adiposity on adult systemic metabolism: a multivariable Mendelian randomization analysis

**DOI:** 10.1093/ije/dyab051

**Published:** 2021-03-30

**Authors:** Tom G Richardson, Juha Mykkänen, Katja Pahkala, Mika Ala-Korpela, Joshua A Bell, Kurt Taylor, Jorma Viikari, Terho Lehtimäki, Olli Raitakari, George Davey Smith

**Affiliations:** 1 MRC Integrative Epidemiology Unit (IEU), Population Health Sciences, Bristol Medical School, University of Bristol, Oakfield House, Oakfield Grove, Bristol, BS8 2BN, UK; 2 Research Centre of Applied and Preventive Cardiovascular Medicine, University of Turku, Turku, Finland; 3 Centre for Population Health Research, University of Turku and Turku University Hospital, Turku, Finland; 4 Paavo Nurmi Centre, Sports and Exercise Medicine Unit, Department of Physical Activity and Health, University of Turku, Turku, Finland; 5 Computational Medicine, Center for Life Course Health Research, Faculty of Medicine, University of Oulu and Biocenter Oulu, Oulu, Finland; 6 NMR Metabolomics Laboratory, School of Pharmacy, University of Eastern Finland, Kuopio, Finland; 7 Department of Medicine, University of Turku and Division of Medicine, Turku University Hospital, Turku, Finland; 8 Department of Clinical Chemistry, Fimlab Laboratories and Finnish Cardiovascular Research Center-Tampere, Faculty of Medicine and Health Technology, Tampere University, Tampere, Finland; 9 Department of Clinical Physiology and Nuclear Medicine, Turku University Hospital, Turku, Finland

**Keywords:** Childhood adiposity, Mendelian randomization, metabolic biomarkers, Young Finns Study, cardiometabolic disease

## Abstract

**Background:**

Individuals who are obese in childhood have an elevated risk of disease in adulthood. However, whether childhood adiposity directly impacts intermediate markers of this risk, independently of adult adiposity, is unclear. In this study, we have simultaneously evaluated the effects of childhood and adulthood body size on 123 systemic molecular biomarkers representing multiple metabolic pathways.

**Methods:**

Two-sample Mendelian randomization (MR) was conducted to estimate the causal effect of childhood body size on a total of 123 nuclear magnetic resonance-based metabolic markers using summary genome-wide association study (GWAS) data from up to 24 925 adults. Multivariable MR was then applied to evaluate the direct effects of childhood body size on these metabolic markers whilst accounting for adult body size. Further MR analyses were undertaken to estimate the potential mediating effects of these circulating metabolites on the risk of coronary artery disease (CAD) in adulthood using a sample of 60 801 cases and 123 504 controls.

**Results:**

Univariable analyses provided evidence that childhood body size has an effect on 42 of the 123 metabolic markers assessed (based on *P* < 4.07 × 10^−4^). However, the majority of these effects (35/42) substantially attenuated when accounting for adult body size using multivariable MR. We found little evidence that the biomarkers that were potentially influenced directly by childhood body size (leucine, isoleucine and tyrosine) mediate this effect onto adult disease risk. Very-low-density lipoprotein markers provided the strongest evidence of mediating the long-term effect of adiposity on CAD risk.

**Conclusions:**

Our findings suggest that childhood adiposity predominantly exerts its detrimental effect on adult systemic metabolism along a pathway that involves adulthood body size.

**Key Messages**

- Children with obesity typically have a higher risk of developing cardiometabolic disease in later life, which can be preceded by metabolic dysfunction. However, there is increasing evidence that lifestyle changes can be enforced to help to mitigate this conferred risk by reducing weight during adolescence.
- In this study, we evaluated whether childhood adiposity has any lasting effect on 123 nuclear magnetic resonance-based measures of systemic metabolism using an approach known as multivariable Mendelian randomization.
- The vast majority of effects between childhood adiposity and circulating metabolites drastically attenuated when accounting for adulthood adiposity (35 out of 42). This suggests that adiposity influences these markers due to a persistent, long-term effect of remaining overweight for many years in life.
- Circulating metabolites related to very-low-density lipoprotein particles provided the strongest evidence of mediating the long-term effect of adiposity on coronary artery disease risk, whereas high-density lipoprotein-related metabolites provided very weak evidence of a mediatory role.
- The biomarkers which showed the strongest evidence of an independent effect of childhood adiposity (amino acids leucine, isoleucine and tyrosine) provided little evidence that they have a downstream influence on disease risk in adulthood.

## Introduction

The rising prevalence of childhood obesity contributes greatly to global healthcare burdens.^1^^,^^2^ Data from the International Childhood Cardiovascular Cohort Consortium suggest that children who are obese who then remain obese as adults have an increased risk of cardiometabolic disease in adulthood. In contrast, children with obesity who do not go on to be obese as adults may have a risk similar to that of non-obese children.^3^ Separating the effects of childhood and adult body size in populations is extremely challenging, however, particularly given that individuals who are overweight during childhood typically remain so as adults.^4^^,^^5^ Furthermore, as diseases such as coronary artery disease (CAD) are preceded by metabolic dysregulation,^6–8^ and because obesity itself is difficult to reduce,^9^ it is also increasingly important to identify molecular biomarkers responsible for mediating effects of adiposity on disease risk.

We recently demonstrated that the challenge of separating effects of adiposity at different life stages can be addressed using human genetics by applying an approach known as multivariable Mendelian randomization (MR).^10–12^ This method exploits the random assortment of genetic alleles within a population to disentangle the effects of multiple closely related exposures (e.g. body size at different life stages) on disease risk. Moreover, under the principles of MR, these genetic variants are inherited quasi-randomly at conception and are thus robust to confounding and reverse causation.^13^^,^^14^

**Figure 1.**
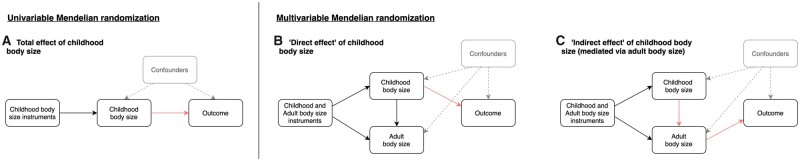
Schematic causal graphs used to illustrate (A) univariable Mendelian randomization (MR) analyses used in this study to estimate total effects between childhood body size and circulating metabolites, (B) multivariable MR analyses to estimate direct effects of childhood body size of circulating metabolites and (C) applying the same multivariable framework to estimate the indirect effects on circulating metabolites mediated along the causal pathway via adult body size. The highlighted arrows on these graphs illustrate the causal effect of childhood body size on the outcome being estimated in MR analyses. The textured arrows and grey shading indicate the effects that MR is typically robust to in comparison to observational analyses.

As illustrated in Figure 1A, MR can be applied in a univariable setting to estimate the effects of childhood body size on complex traits and disease outcomes (e.g. a circulating biomarker or CAD). This is referred to as the ‘total effect’ of child body size, which does not account for adult body size in the model. Previously, we identified strong evidence of a total effect of child body size on adult CAD risk [odds ratio (OR): 1.49 per change in child body size category, 95% confidence interval (CI): 1.33 to 1.68].^10^ Multivariable MR allows the effects of child and adult body size to be simultaneously estimated (Figure 1B and C), making it possible to estimate the ‘direct effect’ of childhood body size that is not mediated via adult body size (Figure 1B). Similarly, the ‘indirect effect’ can also be estimated, which is the contribution mediated along the causal pathway via adult body size (Figure 1C). For example, in the previous analysis on CAD risk, effect estimates from the univariable analysis attenuated to the null when accounting for adult body size (OR: 1.02, 95% CI : 0.86 to 1.22), suggesting that child obesity affects CAD only indirectly via adult obesity. Observational associations between childhood obesity and adult CAD may therefore be explained by individuals remaining obese into adulthood. There is strong support from the literature for this indirect effect on CAD,^15^ although fewer studies have investigated the independent effect of child adiposity on intermediate traits measured in adulthood such as circulating biomarkers of systemic metabolism.

In this study, we aimed to comprehensively estimate the direct and indirect effects of childhood body size on detailed biomarkers of systemic metabolism measured via targeted metabolomics in adulthood,^16^ and the potential role of these biomarkers in mediating risk for CAD. First, we sought to externally validate our derived genetic scores using data from the Young Finns Study (YFS) to reinforce their capability to separate child and adult body size. Next, in two-sample MR, we estimated the total effect of childhood body size on 123 metabolism-related biomarkers using univariable MR. These markers were selected as they were all captured on the nuclear magnetic resonance (NMR) panel analysed in the study by Kettunen *et al.*,^16^ which have been broadly and variably associated with multiple cardiometabolic disease endpoints.^17^ For metabolic markers with the strongest evidence of a genetically predicted effect in this analysis, we applied multivariable MR to examine evidence of direct or indirect effects after accounting for adult body size. We next evaluated which biomarkers may potentially mediate the indirect effect of childhood body size on CAD risk that we found evidence of in our previous work.^10^ Finally, we investigated potential downstream consequences on a wide range of 126 traits and outcomes for markers that may be directly influenced by childhood body size.

## Methods

### Data sources

#### Validation of genetic instruments for childhood and adult body size

We previously identified genetic instruments for childhood and adult body size by undertaking a genome-wide association study (GWAS) of 453 169 individuals of European descent from the UK Biobank study.^18^ Details have been described previously^10^ and are described in detail in Supplementary Note S1 (available as Supplementary data at *IJE* online). In total, there were 295 and 557 genetic instruments detected for childhood and adult body size, respectively, based on conventional genome-wide corrections (i.e. *P* < 5 × 10^−8^) (Supplementary Tables S1 and S2, available as Supplementary data at *IJE* online). Univariable and multivariable evaluations of these instruments provided strong evidence that they are unlikely to suffer from weak instrument bias based on derived F-statistics (Supplementary Table S3, available as Supplementary data at *IJE* online). A comparison of instruments for body size at both time points identified 75 single-nucleotide polymorphisms (SNPs) with *P* < 5 × 10^−8^ at both time points and a comparison of their estimates can be found in Supplementary Table S4 (available as Supplementary data at *IJE* online).

Validation analyses of these genetic instruments were undertaken using measured body mass index (BMI) data from the Cardiovascular Risk in YFS.^19^ Full details of these cohorts can be found in Supplementary Note S2 (available as Supplementary data at *IJE* online).

#### Summary-level data from genome-wide association studies

Summary GWAS data on a total of 123 circulating metabolites measures from NMR quantified in ≤24 925 adults from 14 cohorts (mean age range: 23.9–61.3 years) were available from the Kettunen *et al.* (2016) study^16^ (accessible at http://www.computationalmedicine.fi/data). A high-throughput NMR spectroscopy metabolomics platform was used to quantify the 123 metabolite measures, including lipids and constituents of 14 lipoprotein subclasses (total of 86 measurements), sizes of three lipoprotein particles, two apolipoproteins, 14 fatty acids and their saturation, 9 amino acids, 11 small molecules (involved in glycolysis, citric acid cycle and urea cycle) and 1 inflammatory marker. Details of the NMR metabolomics experimentation and performance have been described previously^16^^,^^20^^,^^21^ and applications in large-scale epidemiological studies have recently been reviewed.^17^ These 123 traits were selected to encompass a broad range of metabolic pathways using a platform originally described by Soininen *et al.*^20^ and have also been broadly and variably associated with multiple cardiometabolic disease.^17^ Summary GWAS data on CAD in a sample of 184 305 (60 801 cases and 123 504 controls) were obtained from the Nikpay *et al.* (2015)^22^ study (accessible at http://www.cardiogramplusc4d.org/data-downloads/).

### Statistical analysis

#### Validating genetic instruments in an external cohort of young Finns

We first evaluated validation of the genetic scores derived from the UK Biobank study that was particularly warranted given that these instruments are based on self-reported recall data. This was undertaken by investigating the capability of both childhood and adult scores to predict obesity in childhood and adulthood using age- and sex-adjusted logistic-regression models in the YFS. Age- and sex-specific international BMI percentiles^23^ were used to extrapolate cut-off points for age 3- to 18-year groups that equate to a BMI of 30 kg/m^2^ in adulthood (Supplementary Table S5, available as Supplementary data at *IJE* online).^3^ The comparison of the UK Biobank categories for body size with those in the YFS can be found in Supplementary Table S6 (available as Supplementary data at *IJE* online). Receiver operating characteristic (ROC) curves were generated for these analyses to determine the area under the curve (AUC) coefficients. Differences in AUC between age- and sex-adjusted logistic-regression models were estimated with the use of the DeLong algorithm.^24^

#### Univariable MR

We applied two-sample MR to estimate the total effect of genetically predicted childhood body size on the 123 circulating biomarkers using statistical packages within the MR-Base platform^25^ (Figure 1A). This was undertaken using the inverse variance weighted (IVW) method, which uses all SNP–outcome estimates regressed on those for the SNP–exposure associations to provide an overall weighted estimate of the causal effect based on the inverse of the square of the standard error for the SNP–outcome association. We applied a conservative Bonferroni correction (i.e. *P* < 0.05/123 = 4.07 × 10^−4^) as a heuristic to allow a manageable number of metabolic biomarkers that are most strongly influenced by genetically predicted childhood body size to be followed up in this study. However, downstream analyses were also repeated on all 123 biomarkers and are included in the Supplementary Materials (available as Supplementary data at *IJE* online) for readers interested in investigating these findings based on a less conservative threshold.

We also undertook various sensitivity analyses in this study to improve the robustness of the findings. This included applying the MR directionality test (also referred to as the ‘Steiger method’) to support evidence that our genetic instrument influences our exposure before our outcome as opposed to the opposite direction of effect.^26^ Moreover, we calculated the intercept term for the MR-Egger method for all univariable analyses to indicate whether directional horizontal pleiotropy may be driving results.^27^

#### Observational effect estimates and comparison with genetic estimates

A linear-regression model was fitted for each variable, with a categorized BMI variable based on the same proportions as those derived in the initial UK Biobank analysis as the explanatory variable and the biomarker measure as the outcome. In the YFS, analyses were performed for those who had data on both childhood/young adulthood BMI and adulthood BMI (*N* = 1508).

#### Multivariable MR

Multivariable MR using the IVW method was subsequently applied in a two-sample setting using the Kettunen *et al.* (2016) circulating metabolites GWAS data. This statistical method fits multiple risk factors as exposures (e.g. childhood and adult body size in this study) to simultaneously estimate their genetically predicted effects on an outcome (e.g. a circulating biomarker). This allowed us to estimate the ‘direct’ effect of childhood body size (i.e. the effect after accounting for adult body size) as well as its ‘indirect’ effect (i.e. the effect mediated by adult body size) on each metabolic biomarker (as depicted in Figure 1B and C). We applied this model using all genetic variants for both childhood and adult body size after undertaking linkage disequilibrium clumping based on *r*^2^ < 0.001 to ensure independence of our instruments. Furthermore, we conducted multivariable MR-Egger analyses to evaluate the horizontal pleiotropy for direct and indirect effects.^28^

#### Evaluating potential downstream consequences on disease outcomes

All circulating biomarkers identified in the initial univariable MR analysis were also further evaluated to determine whether they may mediate the total effect of adiposity on CAD risk. This was undertaken as before using the IVW method and adjusting the resulting *p*-values based on the 123 tests undertaken. For metabolic markers where childhood body size provided evidence of a direct effect based on our multivariable MR analyses based on this conservative threshold, we also evaluated their putative downstream effects in a hypothesis-free manner on 126 diverse traits and disease outcomes curated previously^29^ (Supplementary Table S7, available as Supplementary data at *IJE* online). Our selection criterion in this study was GWASs that had analysed ≥100 000 genetic variants and a study sample size of *n* > 1000, consisting of a population of individuals of European or mixed ancestral descent and who reported all summary statistics necessary to undertake MR analyses. Due to the broad range of disease endpoints that the 123 metabolic markers in this study have been previously associated with, this phenome-wide analysis encompassed a broad range of traits and outcomes.^17^ This included various types of cardiovascular disease [e.g. ischaemic stroke (*n* = 29 633)], autoimmune disease [e.g. inflammatory bowel disease (*n* = 34 652)] and neuropsychiatric diseases [e.g. amyotrophic lateral sclerosis (*n* = 36 052)].^30–32^

All analyses were undertaken using R (version 3.5.1) and SAS (version 9.4). Forest plots were created using the R package ‘ggplot2’.^33^

## Results

### Validation of genetic scores in the YFS

**Figure 2.**
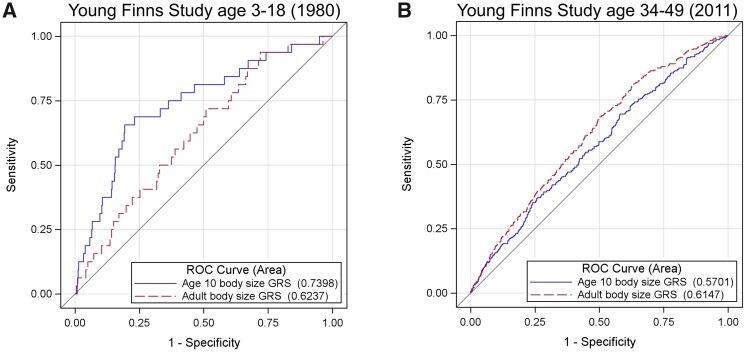
Receiver operator characteristic (ROC) curves to compare the predictive ability of genetic scores for childhood body size (blue) and adult body size (red). (A) ROC curve to investigate the prediction of adiposity during childhood (*N* = 2427, age = 3–18 years) using cut-offs defined in Supplementary Table 4 (available as Supplementary data at *IJE* online) and (B) ROC curve to investigation prediction of adiposity during adulthood based on BMI ≥ 30 kg/m^2^ (*N* = 1762, age = 34–49 years).

The validation study in the YFS demonstrated that our genetic score for childhood body size is a stronger predictor of childhood obesity compared with our adult body-size score [AUCs (95% CI) 0.74 (0.65–0.83) vs 0.62 (0.53–0.72), *P* = 0.02]. Conversely, the adult genetic score was a stronger predictor of adulthood obesity based on a conventional threshold of BMI ≥ 30 kg/m^2^ [0.62 (0.58–0.65)] compared with the childhood score [0.57 (0.54–0.60), *P* = 0.02]. ROC curves illustrating these results can be found in Figure 2. These findings therefore support the utility of these genetic instruments to separate the direct and indirect effects of childhood body size, which builds upon the genetic correlation results reported previously (Supplementary Table S8, available as Supplementary data at *IJE* online). This separation is likely driven by genetic variants that have a statistically larger or smaller magnitude of effect on body size in the original GWAS compared with adulthood (Supplementary Table S9, available as Supplementary data at *IJE* online).

### Evaluating genetic and observational evidence of a total effect between childhood adiposity and systemic metabolism.

Two-sample univariable MR analyses of summary GWAS data provided strong evidence of a total effect between genetically predicted childhood body size and 42 circulating metabolites measured in adulthood (based on *P* < 4.07 × 10^−4^; Supplementary Table S10, available as Supplementary data at *IJE* online). Due to the high correlation that exists between these circulating metabolites, the multiple-testing correction applied in this analysis may be overly stringent; estimates for childhood body size on all 123 markers are therefore plotted in Supplementary Figure S1 (available as Supplementary data at *IJE* online). Results suggested that childhood adiposity has an inverse relationship with high-density lipoprotein (HDL) cholesterol-related markers and a positive relationship with those related to very-low-density lipoprotein (VLDL) cholesterol and triglycerides. There was also strong evidence of a total effect of genetically predicted childhood body size on several amino acids, as well as on glycoprotein acetyls that is a stable marker of cumulative inflammation (Beta = 0.34, SE = 0.06, *P* = 2.83 × 10^−8^). Intercept terms based on the MR-Egger method did not provide strong evidence that horizontal pleiotropy was driving these effects (Supplementary Table S11, available as Supplementary data at *IJE* online) and the MR directionality test supported the direction of effect of childhood body size influencing these circulating biomarkers (Supplementary Table S12, available as Supplementary data at *IJE* online).

**Figure 3.**
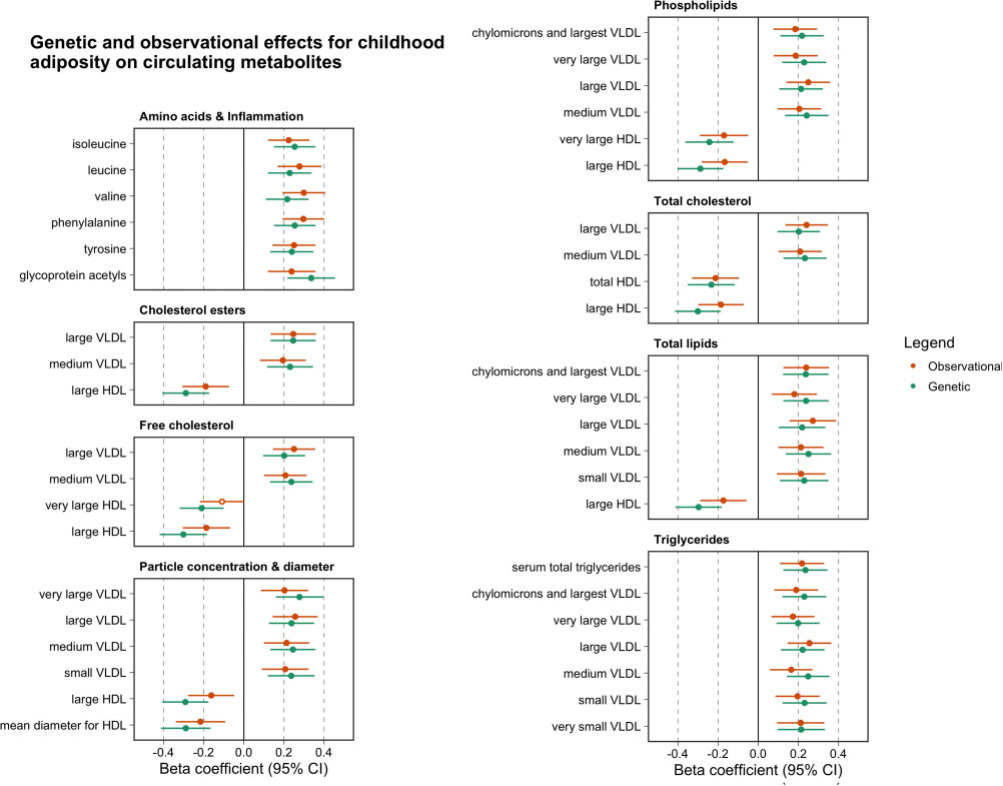
A forest plot depicting the observational (orange) and genetic (green) effect estimates between childhood body size (per change in body size category) and circulating metabolites (per standard deviation unit change). Observational estimates were derived using data from the childhood time point from the Young Finns Study, whereas genetic estimates are based on two-sample Mendelian randomization (MR) analyses using summary data. The observational estimates in this figure have been scaled using a scale factor to have the same magnitude of dispersion around the central estimates as the MR results for comparative purposes.

Observational estimates based on childhood BMI (age 6–12 years) and circulating metabolites based on analyses undertaken in the YFS were comparable to those identified from univariable MR analyses as illustrated in Figure 3 (full results also available in Supplementary Table S13, available as Supplementary data at *IJE* online). Further analyses at the young adult (age 18–24 years) and adult (mean age: 40.2 years, range: 34–46 years) time points in the YFS suggested that the magnitude of effect for BMI on circulating metabolites typically increased over the life course.

### Using multivariable MR to determine whether childhood adiposity has a direct or indirect effect on circulating metabolites.

**Figure 4.**
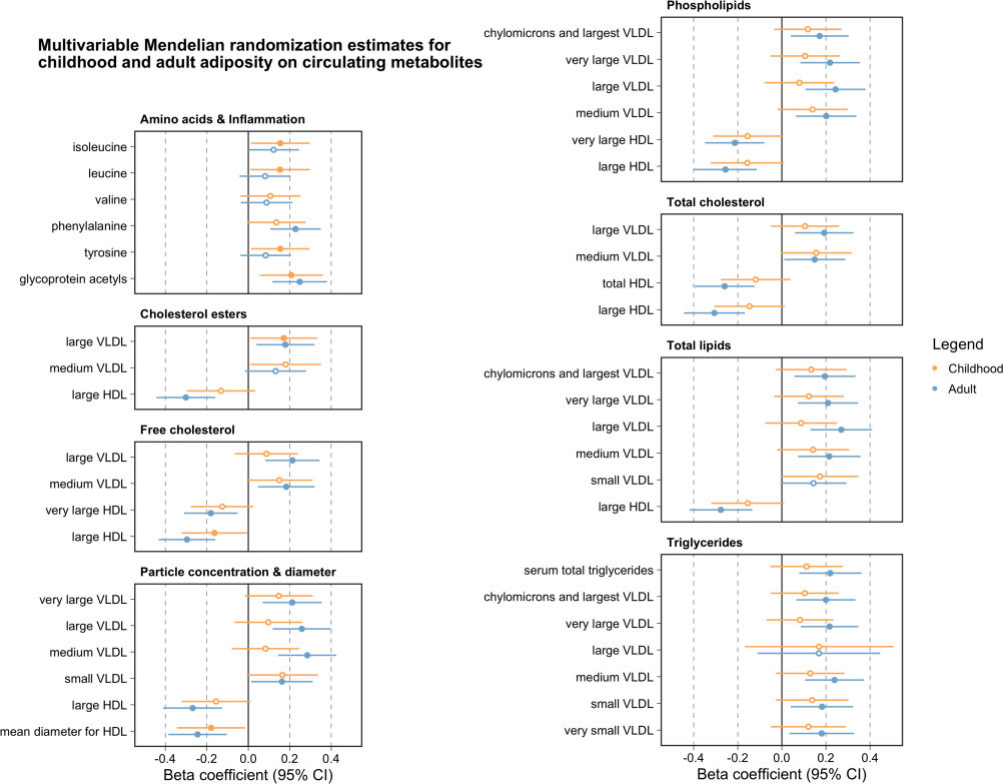
A forest plot illustrating the effect estimates of genetically predicted childhood (yellow) and adult (blue) body size (per change in body size category) on circulating metabolites (per standard deviation change) based on multivariable Mendelian randomization analyses. Points corresponding to estimates whose confidence intervals overlapped with the null were not filled in.

Applying multivariable MR resulted in the majority of effect estimates identified in the previous analysis (35/42) attenuating to include the null upon adjustment for adult body size (Figure 4 and Supplementary Table S14, available as Supplementary data at *IJE* online). This suggests that evidence of a total effect between childhood body size and these metabolic biomarkers, as detected in the univariable analysis, is likely attributed to a long-term persistent effect of adiposity across the life course (i.e. not just during childhood). Of the remaining seven circulating metabolites, the effects of which did not attenuate to the null, there were three biomarkers whose beta effect size for the direct effect of childhood body size was larger in magnitude compared with an indirect effect. These three markers were all amino acids, namely leucine (Beta = 0.15, SE = 0.07, *P* = 0.04), isoleucine (Beta = 0.15, SE = 0.07, *P* = 0.03) and tyrosine (Beta = 0.15, SE = 0.07, *P* = 0.03). Repeating all analyses using the multivariable MR-Egger provided directionally consistent effect estimates to those derived using the IVW method (Supplementary Table S15, available as Supplementary data at *IJE* online). We also undertook IVW MVMR analyses on all remaining 123 circulating metabolites in addition to the 42 that survived the heuristic threshold based on Bonferroni corrections (Supplementary Table S16, available as Supplementary data at *IJE* online).

### Assessing the putative mediatory effects of adiposity-influenced metabolic biomarkers on CAD

**Figure 5.**
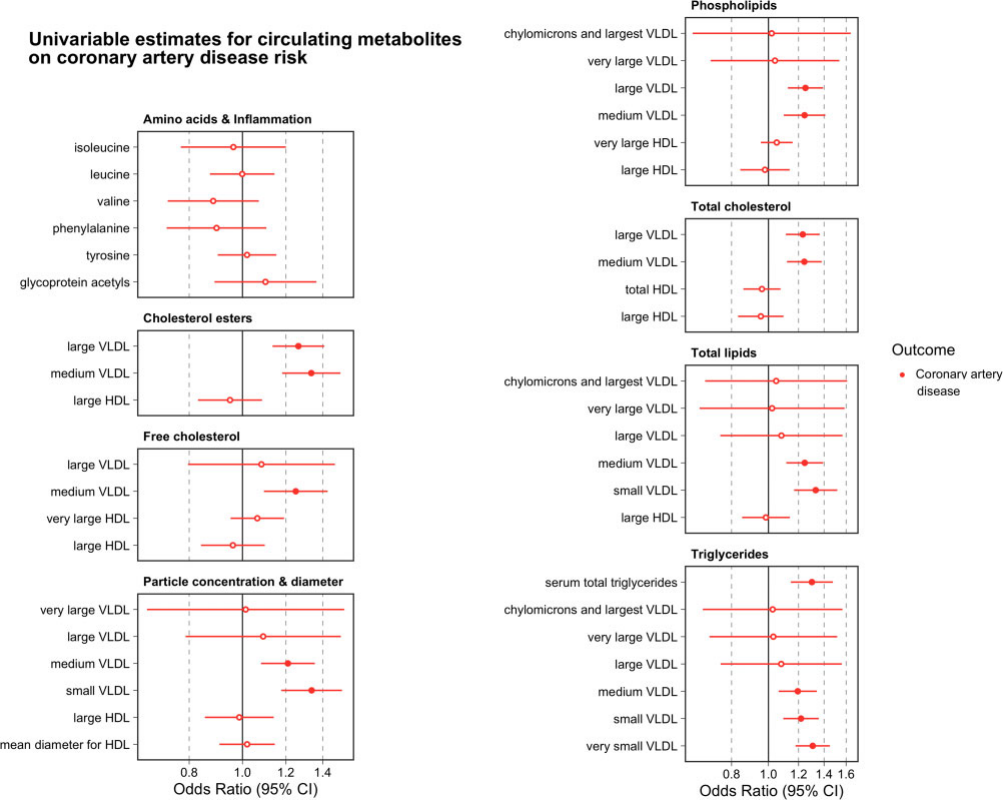
A forest plot illustrating the effect estimates of genetically predicted metabolites (per change in standard deviation increase) on coronary artery disease risk based on univariable Mendelian randomization analyses. Points corresponding to estimates whose confidence intervals overlapped with the null were not filled in.

Univariable MR analyses provided evidence to suggest that 14 of the metabolic markers identified in the initial analysis influence CAD risk at a level of *P* < 0.05/42 = 1.19 × 10^−3^. These were all VLDL-related biomarkers, including serum total triglycerides (OR = 1.30, 95% CI: 1.17 to 1.43, *P* = 5.08 × 10^−5^). As expected, there was a lack of evidence supporting the role of HDL cholesterol-related biomarkers identified in the previous analysis in conferring CAD risk (as is becoming increasingly evident^34^) (Figure 5 and Supplementary Table S17, available as Supplementary data at *IJE* online). There was also no evidence that any of the three amino acids that were highlighted in previous analysis altered CAD risk [leucine (OR = 1.00, 95% CI: 0.86 to 1.13, P = 0.99], isoleucine (OR = 0.96, 95% CI: 0.74 to 1.18, *P* = 0.73) and tyrosine (OR = 1.02, 95% CI: 0.90 to 1.14, *P* = 0.76)). As before, intercept terms using the MR-Egger method did not provide strong evidence that directional horizontal pleiotropy was responsible for these results (Supplementary Table S18, available as Supplementary data at *IJE* online), nor did the MR directionality test indicate that reverse causality was potentially a major issue for these analyses (Supplementary Table S19, available as Supplementary data at *IJE* online). We also repeated univariable MR analyses to estimate the effect of all circulating metabolites (which had at least one genetic instrument based on *P* < 5 × 10^−8^) on CAD risk, not just the 42 that survived conservative Bonferroni corrections (Supplementary Table S20, available as Supplementary data at *IJE* online).

Lastly, we conducted a hypothesis-free analysis for these three amino acids on 126 outcomes (Supplementary Table S7, available as Supplementary data at *IJE* online) to highlight any potential long-term effects they may mediate between childhood body size and later-life disease risk. No results survived multiple-testing corrections for tyrosine or leucine (based on the 126 outcomes analyses i.e. *P* < 0.05/126 = 3.97 × 10^−4^), including outcomes such as breast cancer and anorexia, which may be directly influenced by childhood body size (Supplementary Table S21, available as Supplementary data at *IJE* online). We were only able to instrument isoleucine using a single genetic instrument, although there were 10 outcomes that survived multiple-testing corrections in this analysis (Supplementary Table S22, available as Supplementary data at *IJE* online). However, given that this genetic variant is located at the *GCKR* gene locus, which is known to be highly pleiotropic,^35^^,^^36^ we further evaluated the relationship between childhood and adult body size on these 10 outcomes using multivariable MR as undertaken previously. There was weak evidence that childhood body size has a direct effect on these outcomes (Supplementary Table S23, available as Supplementary data at *IJE* online), which suggests that it is unlikely that circulating isoleucine mediates any putative effect of childhood adiposity on them.

## Discussion

In this study, we investigated the direct and indirect influence of childhood adiposity on 123 circulating biomarkers of systemic metabolism in adulthood. Based on conservative multiple-testing corrections, there was evidence that genetically predicted body size in childhood has a total effect on 42 of these biomarkers in adulthood. However, accounting for adult body size via multivariable MR suggested that such effects of childhood adiposity are mainly indirect, i.e. they are mediated via adult adiposity. Further analyses suggested that several of these biomarkers related to serum triglycerides and VLDL particles that may putatively mediate the effects of adult adiposity on CAD risk. In contrast, there were three amino acids (leucine, isoleucine and tyrosine) that were the only metabolic biomarkers on which childhood adiposity may have a direct effect, although there was no meaningful evidence that these amino acids in turn altered CAD risk.

Leveraging data from large-scale GWAS provides a powerful platform to study causal relationships between modifiable risk factors and disease. Conventionally, however, MR studies have been limited in their application to temporally segmented effects,^37^ which may be attributed in part to the lack of GWAS concerning the onset of and subsequent disease progression.^38^ As such, interpretation of findings in a univariable setting are confined to genetically predicted exposures based on cross-sectional stages in the life course. Recent methodological developments in MR allow multiple exposures to be investigated in a multivariable framework.^11^ Determining whether childhood risk factors have a direct influence on adult disease risk requires modelling them whilst accounting for a measure of the same risk factor taken in adulthood.

Studies from the literature have previously reported strong evidence that BMI causally influences circulating metabolic biomarkers measured in young adulthood^39^ and that such effects of BMI are driven by fat stored centrally.^40^ Results from our univariable analysis of childhood body size further demonstrate this, as there was evidence of a total effect on 42 circulating metabolites based on stringent Bonferroni corrections for multiple testing. In our subsequent multivariable analysis, adjusting analyses for adult body size resulted in 35 of these effects attenuating to the null when accounting for adult body size. This suggests that childhood body size indirectly influences levels of these circulating metabolites via adult body size, as was the case for our previous analysis of CAD.^10^ Corroborating evidence of this cumulative, sustained effect of adiposity on circulating metabolites was identified using observational data from the YFS. In these analyses, we observed that the magnitude of effect for BMI on these circulating metabolites typically increased over the lifespan. Moreover, our results highlight the importance of accounting for adult measures when investigating the effect of early life exposures on later life disease outcome using MR, which is not always conventionally undertaken in the field.^41^^,^^42^ Even when observational studies do account for adult body size, they risk inducing collider bias into their analyses by conditioning on a potential mediator,^43^ which multivariable MR has been shown to be more robust to.^11^

Amongst the 42 circulating metabolites identified in the initial univariable MR analysis, there were 14 biomarkers that may putatively mediate the indirect effect of childhood body size on CAD risk. Notably, these 14 biomarkers were all VLDL- and triglyceride-related. VLDL particles produced by the liver are the major carriers of triglycerides in plasma and are positively associated with both obesity and CAD risk.^44^^,^^45^ A recent study also supports evidence that VLDL metabolic markers mediate a substantial component of the effect of obesity on myocardial infarction risk,^46^ although further research is required to evaluate what proportion of this effect triglyceride particles are responsible for. Conversely, there was a lack of evidence from downstream analyses that any HDL-related measure identified in initial analyses influence CAD risk. These findings therefore corroborate evidence from various studies and trial outcomes that support HDL cholesterol or apolipoprotein A-I as non-causal for CAD,^47–50^ although it may still be useful for risk prediction.^51^^,^^52^

Our multivariable analysis also suggested that only 3 of the 42 circulating metabolites highlighted by our univariable analyses may putatively be influenced directly by childhood body size. These were the amino acids leucine, isoleucine and tyrosine. Each of these biomarkers has been associated with obesity and cardiometabolic health in previous studies,^53–55^ although our analysis suggests that childhood body size may directly influence their levels, potentially in addition to adult body size. However, we found no strong evidence in our study to suggest that these direct effects would have downstream consequences on CAD risk in adulthood.

Our study has several limitations that should be taken into account when interpreting the results. The childhood body-size instruments used were derived using recall-questionnaire data, which is why we have undertaken analyses in the YFS cohort to provide additional evidence of validation. That said, future GWASs of measured childhood adiposity will be preferable to this score once large-scale sample sizes are available, although, for the time being, our scores have been derived in a sample size far larger than any study of measured childhood adiposity. This score has also recently been shown to be a stronger predictor of childhood BMI compared with adulthood-measured scores in the HUNT study in Norway as well as the YFS.^56^ Additionally, future data sets will likely facilitate analyses assessing the impact of weight change over the life course on disease risk to be investigated, which may potentially identify evidence of effects independently of childhood and adult body size. Likewise, our genetic scores are based on body size at specific time points [i.e. prepuberty and mid-adulthood (mean age ∼55 years)], which therefore lack the precision to identify critical windows throughout the lifecourse where the effect of adiposity on disease begins to become immutable. For example, there is previous evidence to suggest that being overweight in late adolescence may still increase the risk of CAD even after adjustment for adulthood BMI.^57^ Furthermore, we note that our body-size scores do not differentiate between fat and lean mass, which is particularly important to take into account for the branched-chain amino acids analysed in this study such as leucine and isoleucine.

We also acknowledge that, although the premise of MR is to use genetic instruments as proxies to mimic variation in modifiable risk factors, the genetically predicted body size may not directly equate to weight change due to lifestyle changes such as diet or exercise as discussed in the early MR papers with respect to the gene–environment equivalence assumption of MR elsewhere.^58^ Furthermore, the 123 circulating metabolites analysed in this study are predominantly based on lipoprotein lipids, which leaves scope to expand upon our analyses in the future, particularly given that the number of metabolites with GWAS data has been expanded upon by recent work.^59^ Lastly, we have only used metabolic data from one source in this work for MR analyses due to the availability of GWAS summary statistics. Therefore, replication of our results is warranted when those data become accessible.

In conclusion, our findings suggest that the influence of early life adiposity on adult systemic metabolism is predominantly due to an indirect pathway via adulthood body size. Atherogenic VLDL particles may further mediate these effects of sustained adult adiposity on adult CAD risk, whereas evidence of a mediatory role was not supported for amino acids or HDL particles. The impact of childhood obesity on adult cardiometabolic disease risk may therefore be mitigated by reducing adult adiposity or by targeting intermediate traits like triglyceride-rich lipoproteins if such reductions are infeasible.

## Supplementary data

Supplementary data are available at *IJE* online.

## Ethics approval

Ethical approval for the YFS was approved by local committees and participants gave written informed consent. All other analyses were conducted using summary-level data generated by previous studies that have described their relevant ethical approvals.

## Funding

This work was supported by the Integrative Epidemiology Unit, which receives funding from the UK Medical Research Council and the University of Bristol [MC_UU_00011/1]. GDS conducts research at the NIHR Biomedical Research Centre at the University Hospitals Bristol NHS Foundation Trust and the University of Bristol. The views expressed in this publication are those of the author(s) and not necessarily those of the NHS, the National Institute for Health Research or the Department of Health. T.G.R. is a UKRI Innovation Research Fellow [MR/S003886/1]. J.A.B. is supported by the Elizabeth Blackwell Institute for Health Research, University of Bristol and the Wellcome Trust Institutional Strategic Support Fund [204813/Z/16/Z]. K.T. is supported by a British Heart Foundation Doctoral Training Program [FS/17/60/33474]. The Special Turku Coronary Risk Factor Intervention Project study is funded by the Academy of Finland [grants 206374, 294834, 251360, 275595 and 322112], the Juho Vainio Foundation, the Finnish Foundation for Cardiac Research, the Finnish Ministry of Education and Culture, the Finnish Cultural Foundation, the Sigrid Jusélius Foundation, Special Governmental Grants for Health Sciences Research (Turku University Hospital), the Yrjö Jahnsson Foundation and the Turku University Foundation. The Young Finns Study is funded by the Academy of Finland [grants 286284, 134309 (Eye), 126925, 121584, 124282, 129378 (Salve), 117787 (Gendi), 41071 (Skidi) and 322098 (for T.L.)]; the Social Insurance Institution of Finland; Competitive State Research Financing of the Expert Responsibility area of Kuopio, Tampere and Turku University Hospitals [grant X51001]; Juho Vainio Foundation; Paavo Nurmi Foundation; Finnish Foundation for Cardiovascular Research; Finnish Cultural Foundation; The Sigrid Jusélius Foundation; Tampere Tuberculosis Foundation; Emil Aaltonen Foundation; Yrjö Jahnsson Foundation; Signe and Ane Gyllenberg Foundation; Diabetes Research Foundation of Finnish Diabetes Association; and EU Horizon 2020 [grant 755320 for TAXINOMISIS and grant 848146 To-Aition]; and European Research Council [grant 742927 for MULTIEPIGEN project]; Tampere University Hospital Supporting Foundation. M.A.K. is funded by a research grant from the Sigrid Jusélius Foundation, Finland.

## Data availability

Further information regarding data from the Young Finns Study, as well as proposals for collaboration and data access, can be requested by contacting Prof. Olli Raitakari. Genetic instruments on childhood and adult body size were obtained from the previous study by Richardson *et al.* (2020) using data from the UK Biobank study (https://www.ukbiobank.ac.uk/enable-your-research/apply-for-access). Metabolites GWAS data are publicly available at http://www.computationalmedicine.fi/data/NMR_GWAS/. Publicly available GWAS data on coronary artery disease were obtained from http://www.cardiogramplusc4d.org/data-downloads/. All other GWAS data are publicly available at https://gwas.mrcieu.ac.uk/.

## Supplementary Material

dyab051_Supplementary_Data
