# Supplementary material for: Evaluating the direct effects of childhood adiposity on adult systemic metabolism: a multivariable Mendelian randomization analysis
Source: Int J Epidemiol. 2021 Mar 30;50(5):1580–92. doi: 10.1093/ije/dyab051 (PMC8580280; doi:10.1093/ije/dyab051)
Supplement: dyab051_Supplementary_Data [file dyab051_supplementary_data.zip › ije-2020-08-1617-File008.docx]

**Supplementary Note S1**

## Identification of genetic instruments for childhood and adult body size

Full details of how these instruments were derived can be found in the publication by Richardson et al (Richardson *et al.*, 2020). In brief, we used questionnaire data asking participants ‘When you were 10 years old, compared to average would you describe yourself as thinner, plumper or about average?’ to define our childhood body size variable. We then harmonized adult measured body mass index (BMI) by converting it into a categorical variable with 3 groups based on the same proportions as the childhood measure (i.e. ‘thinner’, ‘plumper’ and ‘about average’).

GWAS in the UK Biobank study are particularly susceptible to population structure as demonstrated elsewhere (Abdellaoui *et al.*, 2019). We therefore undertook analyses adjusted for age, sex and genotyping chip using the BOLT-LMM software which generates a genetic relationship matrix between samples to account for relatedness and population stratification (Loh *et al.*, 2015).

We previously attempted to validate these findings by comparing their genetic correlations with results from GWAS of measured childhood obesity (Bradfield *et al.*, 2012) and adult BMI analysed as a continuous variable (Speliotes *et al.*, 2010). Despite the use of recall data in our study to derive the childhood body size instruments, we found that our results were more strongly correlated with measured childhood obesity (r_g_=0.85) compared to adult BMI (rg=0.67). In contrast, our adult body size GWAS was very strongly correlated with the adult BMI findings (rg=0.96) in comparison to their correlation with childhood obesity (rg=0.64). We also previously evaluated the prediction of these scores on measured BMI at three timepoints from the Avon Longitudinal Study of Parents and Children (ALSPAC)(Boyd *et al.*, 2019). As illustrated below, we found that childhood score most strongly predicts childhood BMI at age 9 in ALSPAC, whereas the adult score is the strongest predictor of BMI in adulthood:

**
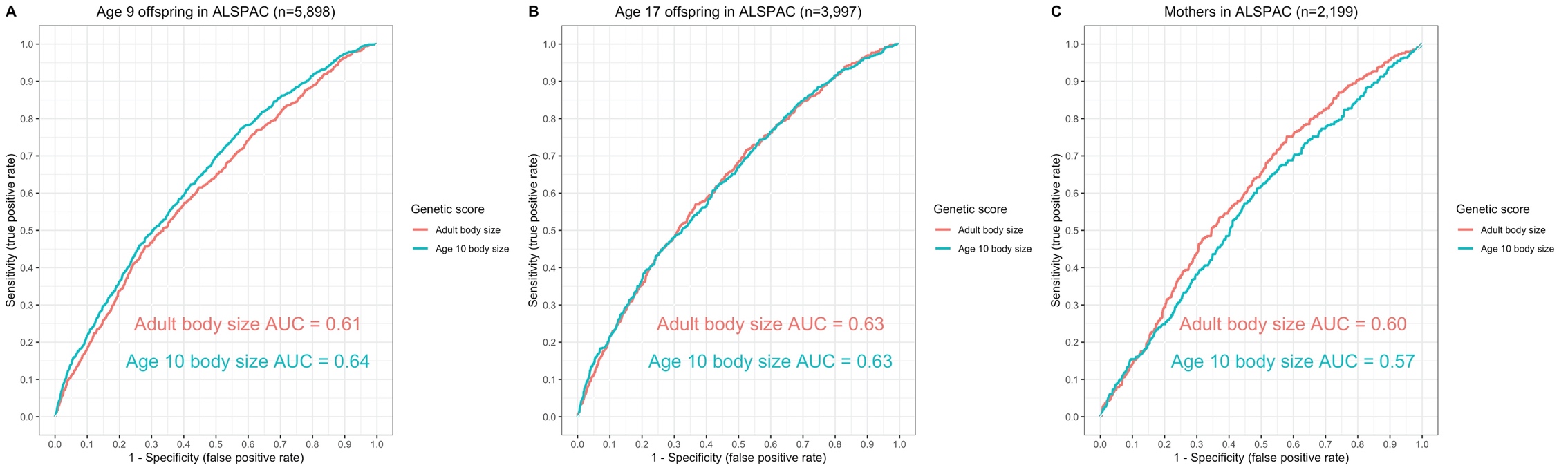
**

The genetic risk scores constructed in this study were also previously constructed in the Avon Longitudinal Study of Parents and Children (ALSPAC) (Richardson et al (2020)). Area under the curve (AUC) plots were generated based on three timepoints in ALSPAC, defining obesity as individuals with a body mass index ≥ the 85^th^ percentile.

**Supplementary Note S2**

***The Cardiovascular Risk in Young Finns Study***

YFS is a multicenter follow-up study in five cities and their rural surroundings in Finland to evaluate atherosclerotic risk factors from childhood into adulthood. The study began in 1980 when 3,596 participants randomly selected from the national register were examined (boys and girls; ages 3, 6, 9, 12, 15, and 18 years). Subsequently, follow-up studies have been conducted regularly. Here, BMI data collected at 1980, 1983, 1986 (from childhood to young adulthood) and 2011 (adulthood) follow-up studies were included. Metabolic traits derived using nuclear magnetic resonance (NMR) spectroscopy were available from the adulthood timepoint in YFS. Local ethics committees approved the study and participants gave written informed consent. Details of the study design have been presented previously (Raitakari *et al.*, 2008).

Adiposity variables

Height and weight were measured and BMI was calculated as weight in kilograms over height in meters squared. YFS follow-up cohort data was used to define obesity in childhood at 3 to 18 years of age using age specific and sex specific BMI cutoff points (1). Adulthood obesity was defined by BMI ≥ 30. To utilize all available repeatedly measured data, the area under the curve (AUC) for continuous BMI was evaluated to indicate a long-term exposure of BMI (2). Subject-specific curves for BMI were estimated by mixed model regression splines (3). For this study, the BMI AUC variables were defined separately for childhood (6 to 12 years) and young adulthood (18 to 24 years) for participants with at least two BMI measures from the aforementioned age periods. To facilitate comparison with the UK Biobank BMI variables, the continuous YFS BMI variables (childhood AUC, young adulthood AUC and adulthood) were converted into a categorical variables with 3 groups (‘thinner’, ‘plumper’ and ‘about average’) based on the same proportions as in (4).

Nuclear magnetic resonance (NMR) metabolomics

A high-throughput NMR platform was used to quantify serum metabolites of YFS (2011 follow-up study, N=2040). Details of the NMR method have been presented earlier (6), and NMR-based profiling has been used in various epidemiological studies (7). Serum NMR-based variables with skewed distributions were log-transformed prior to statistical analyses.

Genotyping, Genotype Imputation and construction of genetic risk scores

Genotyping in YFS was performed by using a custom-made Illumina Human 670 K BeadChip and genotype imputation for 2,442 samples was performed with the 1000G Phase I Integrated Release Version 3 as a reference panel, as described in more detailed in (8). Separate weighted genetic risk scores (GRSs) for childhood BMI comprising 277 SNPs and for adulthood BMI comprising 516 SNPs were calculated as a sum of genotyped risk alleles or imputed allele dosages carried by an individual, each multiplied by the effect size (beta) reported in (4). 18 SNPs associated with childhood BMI and 41 SNPs associated with adulthood BMI in UK Biobank were not available among genotyped or imputed SNPs of YFS. Lists of SNPs and effect sizes used in the wGRS calculations are provided in **Supplementary Table S1**.

**Supplementary Figure S1**


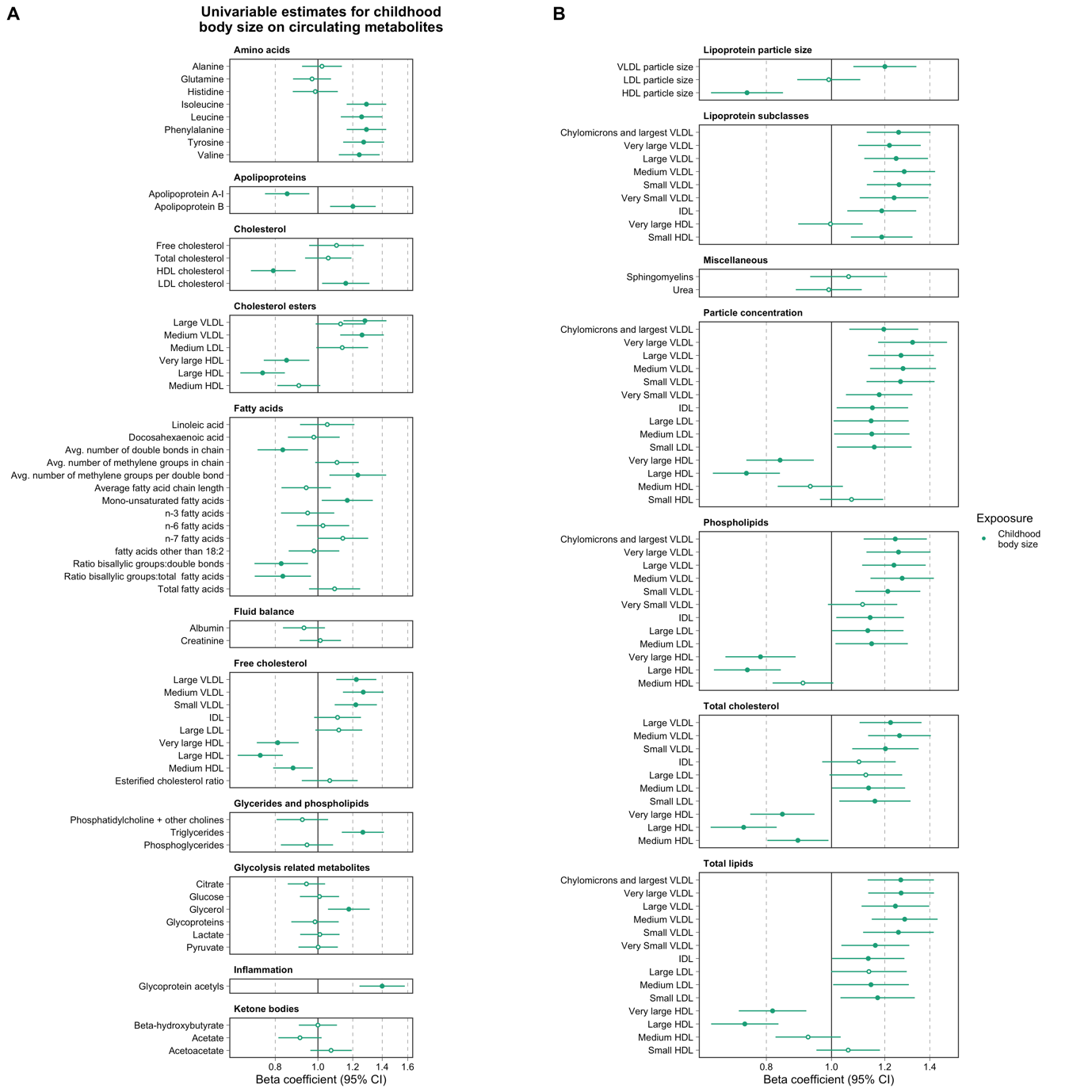


A forest plot illustrating the univariable Mendelian randomization estimate for the total effect of childhood body size on 123 circulating metabolites.

**References**

Abdellaoui, A., Hugh-Jones, D., Yengo, L., Kemper, K. E., Nivard, M. G., Veul, L., Holtz, Y., Zietsch, B. P., Frayling, T. M., Wray, N. R., Yang, J., Verweij, K. J. H. & Visscher, P. M. 2019. Genetic correlates of social stratification in Great Britain. *Nat Hum Behav,* 3**,** 1332-1342.

Boyd, A., Thomas, R., Hansell, A. L., Gulliver, J., Hicks, L. M., Griggs, R., Vande Hey, J., Taylor, C. M., Morris, T., Golding, J., Doerner, R., Fecht, D., Henderson, J., Lawlor, D. A., Timpson, N. J. & Macleod, J. 2019. Data Resource Profile: The ALSPAC birth cohort as a platform to study the relationship of environment and health and social factors. *Int J Epidemiol,* 48**,** 1038-1039k.

Bradfield, J. P., Taal, H. R., Timpson, N. J., Scherag, A., Lecoeur, C., Warrington, N. M., Hypponen, E., Holst, C., Valcarcel, B., Thiering, E., Salem, R. M., Schumacher, F. R., Cousminer, D. L., Sleiman, P. M., Zhao, J., Berkowitz, R. I., Vimaleswaran, K. S., Jarick, I., Pennell, C. E., Evans, D. M., St Pourcain, B., Berry, D. J., Mook-Kanamori, D. O., Hofman, A., Rivadeneira, F., Uitterlinden, A. G., van Duijn, C. M., van der Valk, R. J., de Jongste, J. C., Postma, D. S., Boomsma, D. I., Gauderman, W. J., Hassanein, M. T., Lindgren, C. M., Magi, R., Boreham, C. A., Neville, C. E., Moreno, L. A., Elliott, P., Pouta, A., Hartikainen, A. L., Li, M., Raitakari, O., Lehtimaki, T., Eriksson, J. G., Palotie, A., Dallongeville, J., Das, S., Deloukas, P., McMahon, G., Ring, S. M., Kemp, J. P., Buxton, J. L., Blakemore, A. I., Bustamante, M., Guxens, M., Hirschhorn, J. N., Gillman, M. W., Kreiner-Moller, E., Bisgaard, H., Gilliland, F. D., Heinrich, J., Wheeler, E., Barroso, I., O'Rahilly, S., Meirhaeghe, A., Sorensen, T. I., Power, C., Palmer, L. J., Hinney, A., Widen, E., Farooqi, I. S., McCarthy, M. I., Froguel, P., Meyre, D., Hebebrand, J., Jarvelin, M. R., Jaddoe, V. W., Smith, G. D., Hakonarson, H., Grant, S. F. & Early Growth Genetics, C. 2012. A genome-wide association meta-analysis identifies new childhood obesity loci. *Nat Genet,* 44**,** 526-31.

Loh, P. R., Tucker, G., Bulik-Sullivan, B. K., Vilhjalmsson, B. J., Finucane, H. K., Salem, R. M., Chasman, D. I., Ridker, P. M., Neale, B. M., Berger, B., Patterson, N. & Price, A. L. 2015. Efficient Bayesian mixed-model analysis increases association power in large cohorts. *Nat Genet,* 47**,** 284-90.

Raitakari, O. T., Juonala, M., Ronnemaa, T., Keltikangas-Jarvinen, L., Rasanen, L., Pietikainen, M., Hutri-Kahonen, N., Taittonen, L., Jokinen, E., Marniemi, J., Jula, A., Telama, R., Kahonen, M., Lehtimaki, T., Akerblom, H. K. & Viikari, J. S. 2008. Cohort profile: the cardiovascular risk in Young Finns Study. *Int J Epidemiol,* 37**,** 1220-6.

Richardson, T. G., Sanderson, E., Elsworth, B., Tilling, K. & Davey Smith, G. 2020. Use of genetic variation to separate the effects of early and later life adiposity on disease risk: mendelian randomisation study. *BMJ,* 369**,** m1203.

Speliotes, E. K., Willer, C. J., Berndt, S. I., Monda, K. L., Thorleifsson, G., Jackson, A. U., Lango Allen, H., Lindgren, C. M., Luan, J., Magi, R., Randall, J. C., Vedantam, S., Winkler, T. W., Qi, L., Workalemahu, T., Heid, I. M., Steinthorsdottir, V., Stringham, H. M., Weedon, M. N., Wheeler, E., Wood, A. R., Ferreira, T., Weyant, R. J., Segre, A. V., Estrada, K., Liang, L., Nemesh, J., Park, J. H., Gustafsson, S., Kilpelainen, T. O., Yang, J., Bouatia-Naji, N., Esko, T., Feitosa, M. F., Kutalik, Z., Mangino, M., Raychaudhuri, S., Scherag, A., Smith, A. V., Welch, R., Zhao, J. H., Aben, K. K., Absher, D. M., Amin, N., Dixon, A. L., Fisher, E., Glazer, N. L., Goddard, M. E., Heard-Costa, N. L., Hoesel, V., Hottenga, J. J., Johansson, A., Johnson, T., Ketkar, S., Lamina, C., Li, S., Moffatt, M. F., Myers, R. H., Narisu, N., Perry, J. R., Peters, M. J., Preuss, M., Ripatti, S., Rivadeneira, F., Sandholt, C., Scott, L. J., Timpson, N. J., Tyrer, J. P., van Wingerden, S., Watanabe, R. M., White, C. C., Wiklund, F., Barlassina, C., Chasman, D. I., Cooper, M. N., Jansson, J. O., Lawrence, R. W., Pellikka, N., Prokopenko, I., Shi, J., Thiering, E., Alavere, H., Alibrandi, M. T., Almgren, P., Arnold, A. M., Aspelund, T., Atwood, L. D., Balkau, B., Balmforth, A. J., Bennett, A. J., Ben-Shlomo, Y., Bergman, R. N., Bergmann, S., Biebermann, H., Blakemore, A. I., Boes, T., Bonnycastle, L. L., Bornstein, S. R., Brown, M. J., Buchanan, T. A., et al. 2010. Association analyses of 249,796 individuals reveal 18 new loci associated with body mass index. *Nat Genet,* 42**,** 937-48.
